# Workplace bullying and sexual harassment at work among hotel housekeepers in the Balearic Islands (Spain)

**DOI:** 10.3389/fpsyg.2023.1241255

**Published:** 2024-01-09

**Authors:** Xenia Chela-Alvarez, Oana Bulilete, M. Esther García-Buades, Victoria A. Ferrer-Perez, Joan Llobera

**Affiliations:** ^1^Primary Care Research Unit of Mallorca, Balearic Islands Health Service, Palma, Spain; ^2^GrAPP-caIB – Health Research Institute of the Balearic Islands (IdISBa), Palma, Spain; ^3^RICAPPS- Red de InvestigaciónCooperativa de Atención Primaria y Promoción de la Salud – Carlos III Health Institute (ISCIII), Madrid, Spain; ^4^Department of Psychology, University of the Balearic Islands, Palma, Spain

**Keywords:** sexual harassment at work, workplace bullying, hotel housekeepers, self-rated health, mixed-methods study, job satisfaction, wage satisfaction, job stress

## Abstract

**Introduction:**

Hotel housekeepers are close to being a 100% feminized occupational group in Spain. This fact, coupled with some features of the job, places them at high risk of sexual harassment at work and bullying in the workplace. This study aims to explore experiences of sexual harassment at work and workplace bullying among hotel housekeepers in the Balearic Islands. Second, it aims to describe and estimate the prevalence of both phenomena.

**Methods:**

This is a mixed-methods study. Ten semi-structured interviews were conducted with key informants, and six focus groups were held with hotel housekeepers. Additionally, a quantitative cross-sectional study (*n* = 1,043) was undertaken.

**Results:**

Most participants in focus groups had been sexually harassed at work. However, they had normalized this kind of situations, not labeling themselves as victims of sexual harassment; and harassment events were seen as unimportant, normal, and unquestioned, as well as being part of their daily work. Hotel housekeepers who were sexually harassed indicated high levels of stress at work and low social support. The prevalence of different workplace bullying behaviors was quite high among hotel housekeepers working in the Balearic Islands. Some were associated with poorer self-rated health, less satisfaction with the job and the salary, lower social support, and higher levels of stress. Despite this, qualitative methods informed us that less severe behaviors were normalized and perceived by hotel housekeepers as intrinsic to their job.

**Discussion:**

The results show the high tolerance to less severe expressions of sexual harassment at work and workplace bullying, as well as difficulties in or reluctance to labeling this kind of experiences as such.

## 1 Introduction

### 1.1 Justification, objectives, and benefits

Sexual harassment at work (SHaW) and workplace bullying (WB) constitute workplace injustice or discrimination and are a workplace health and safety problem (Campbell and McFadyen, 2017). In addition, SHaW and WB undermine equality at work and constitute an attack on civil and human rights (ILO, 2020).

SHaW and WB occur in many occupational settings and economic sectors, including the tourism sector. The tourism sector makes an important contribution to the Spanish economy. In 2021, it was estimated to represent 8.0% of the gross domestic product (INE, 2021). In the Balearic Islands, 13.2% of the employed population was in the accommodation services (3rd quarter of 2022). It is estimated that approximately 13,000 hotel housekeepers (HHs) work in the Balearic Islands. HHs, mostly women, are mainly in charge of cleaning guests’ rooms and common areas (i.e., the lobby). Hotel housekeeping is a precarious job because most HHs work in a recurring-seasonal manner, which implies not having a stable income throughout the year. Furthermore, the combination of feminization of the job and low job status has been directly related to their vulnerability to harassment (Hoel and Einarsen, 2003).

Most definitions of sexual harassment are based on Mackinnons’ work, for whom “sexual harassment (…) refers to the unwanted imposition of sexual requirements in the context of a relationship of unequal power”(MacKinnon, 1979:1). Although sexual harassment can affect anyone, it particularly affects women (United Nation Women, 2018; International Labour Organization (ILO), 2020). The International Labor Organization (ILO) describes two key elements in the definition of SHaW. The first one, the *quid pro quo* element, refers to any kind of conduct of a sexual nature that is unwelcome and offensive to the recipient and is used to make work-related decisions. The second one is that this kind of conduct creates a hostile work environment for the recipient but also for the witnesses (Hauge et al., 2007; International Labour Organization (ILO), 2020) and negatively impacts recipients’ health (Okechukwu et al., 2014; Hershcovis et al., 2016). Behaviors and actions involving sexual harassment include verbal comments and dirty jokes, sexual gestures, and touching. Furthermore, SHaW includes behaviors coming from those in positions of authority, subordinates, or clients (Fitzgerald and Cortina, 2018).

Even though SHaW is a problem that has been studied over the last few decades, its actual prevalence remains unknown (Fitzgerald and Cortina, 2018), and only estimations are available. Several meta-analyses and population-based studies recently carried out estimated the prevalence of sexual harassment or violence at workplaces (Zeng et al., 2019; Basile et al., 2020; Lu et al., 2020; Worke et al., 2020; Ranganathan et al., 2021). The prevalence differed among studies, depending above all on the geographical context (north vs. middle-and low-income countries), the occupational group, and the method or questions used, as specifically indicated in Ilies et al. (2003). Morgan and Pritchard (2019) stated that SHaW is especially prevalent in the hospitality sector. Data from the ‘Survey on violence against women in the European Union’ (European Union Agency for Fundamental Rights, 2012) showed that among European women who had experienced sexual harassment at least once since the age of 15, about 32% reported the perpetrator to be somebody in their job context; this percentage was 20% in the case of Spain. More current data from the Spanish mega-survey of ‘Violence against women’ (Subdirección General de Sensibilización, 2020) revealed that out of the women who had suffered sexual harassment and answered the question about who the perpetrator was, about 17.3% declared to have been sexual harassed by a man from their work and 1.1% by a woman from their work. Additionally, out of the women who suffered stalking—when the harassment is repeated—4.1% was by their boss and 7.3% was by a man at their workplace (Subdirección General de Sensibilización, 2020). The rest were harassed by an unknown person or a person from a non-work context.

Hotel settings have been identified as one of the settings with most cases SHaW and WB (Milczarek, 2010). The #Metoo movement, which spread globally in October 2017 (Ram, 2021) and in which people shared their experiences of sexual harassment through social networks, raised the visibility of sexual harassment in hospitality settings. In the tourism industry, HHs can be harassed by guests, supervisors/managers, and/or co-workers. Hotels’ recommendations to HHs to leave the door open while cleaning and tidying acknowledge HHs’ vulnerability to guest-initiated sexual harassment (Nimri et al., 2020).

### 1.2 Theoretical and previous findings

#### 1.2.1 Sexual harassment at work

Some characteristics of the hotel housekeeping job explain HHs’ vulnerability to sexual harassment by guests, such as working alone, away from busy and common spaces (i.e., the lobby), and interacting with customers (Hunter and Watson, 2006; ILO, 2017; Mensah, 2022). HHs usually clean the room when guests are not present, but sometimes they find somebody inside the room or guests come in while they are working (Hunter and Watson, 2006). These characteristics, coupled with the fact that guests are away from home and away from their daily constraints, might increase the likelihood of unethical tourist behaviors (Hunter and Watson, 2006). One study found that 44 out of 46 HHs experienced sexual approaches by male guests, many being international guests (Kensbock et al., 2015).

Kensbock et al. (2016) reflect on two characteristics of the HHs’ job encouraging SHaW: being female and performing a traditional role. Uniform style was a way to sexualize HHs, a fact perceived by HHs as encouraging guests to sexually harass them (Kensbock et al., 2016). Additionally, the tasks involved are associated with domestic work, underscoring women’s traditional roles; housekeeping is considered unskilled work, because the skills needed are conceived to be inherent to females, and it has been labeled as ‘dirty work’ (Nimri et al., 2020). In this line, Kensbock et al. (2016) reported HHs’ perceptions of inferiority and invisibility. Overall, these locate HHs on two axes of inequality or discrimination: being women and having a low socioeconomic status (Kensbock et al., 2016), thereby facing structure-related violence not only from their supervisors but also from guests and male co-workers (Ram, 2018).

The results of several meta-analyses highlighted that organizational factors are more important than individual ones to predict SHaW (Ilies et al., 2003; Willness et al., 2007; Cantisano et al., 2008). Organizational factors include “men being numerically, structurally and stereotypically dominant”(Cortina and Areguin, 2021; p. 295); organizational climate of tolerance toward sexual harassment behaviors; and masculinity contest cultures—characterized by hard competition, disdain for personal relationships, displays of strength, etc. (Fitzgerald and Cortina, 2018; Cortina and Areguin, 2021).

Outcomes of SHaW include the creation of a hostile work environment, personal suffering, damage to the victim’s reputation, the victim’s loss of dignity and self-esteem, and blaming the victim’s behaviors by relatives, friends, and peers (ILO, 2020). Some health consequences associated with SHaW are neck pain (Stock and Tissot, 2012), psychological distress (Jung and Yoon, 2020), and post-traumatic disorder symptoms (Ho et al., 2012). Even less intense (but frequent) forms of SHaW have been identified as decreasing the victim’s wellbeing (Sojo et al., 2016). SHaW also entails experiencing more stress at work (Leskinen et al., 2011), increasing job and co-worker dissatisfaction (Willness et al., 2007; Leskinen et al., 2011; Merkin and Shah, 2014), and negative economic consequences for the victim, including changing their job, reducing working hours, financial losses, and a negative impact on their career progression (Fitzgerald and Cortina, 2018; McLaughlin et al., 2018; ILO, 2020) SHaW also involves economic costs for the organization; it negatively affects its functioning (ILO, 2020), increasing worker burnout (Jung and Yoon, 2020), turnover intentions (Ilies et al., 2003; Willness et al., 2007; Cantisano et al., 2008), and absenteeism (Merkin and Shah, 2014).

#### 1.2.2 Workplace bullying

There is no consensus regarding the definition of WB. Despite this, Einarsen et al. (2003) defined WB as “the systematic mistreatment of a subordinate, a colleague, or a superior, which, if continued, may cause severe social, psychological, and psychosomatic problems in the victim.” WB includes behaviors such as assigning unpleasant tasks to the victim; excluding or ignoring them at work; and insulting them or spreading rumors about them (Hershcovis et al., 2016).

Not only is the frequency and duration of the situation important in order for it to be labeled as WB, but also in this situation the victim is unable or has difficulties in defending themselves (Leymann, 1990, 1996; Einarsen and Skogstad, 1996). One systematic review reported that women were more likely to suffer WB than men (Feijó et al., 2019).

The predominant framework explaining the antecedents of WB is the *work environmental hypothesis*, which states that stressful and poorly organized work environments might lead to conditions that make bullying situations emerge (Milczarek, 2010; Hershcovis et al., 2016; Feijó et al., 2019). Accordingly, previous empirical evidence suggests that the main factors associated with WB among hospitality employees were related to working conditions (Bohle et al., 2017; Ariza-Montes et al., 2018). Ariza-Montes et al. (2018) found that these factors were working at high speed, the perception that one’s health was at risk because of work, dissatisfaction with working conditions, and interacting with angry customers. The results by Bohle et al. (2017) indicated that disorganization and regulatory failure were positively related to WB.

Health-related and organizational outcomes have been described among hospitality workers who had suffered from WB. Several studies reported that WB was negatively related to employees’ wellbeing (Ram, 2018; Hsu et al., 2019; Hayat and Afshari, 2021) and positively associated with emotional exhaustion (Srivastava and Agarwal, 2020). These studies also found that organizational factors (i.e., perceived organizational support and organizational justice) reduced the negative effect of WB on wellbeing. At the organizational level, an increase in burnout and higher intentions to quit have been described (Bohle et al., 2017; Ram, 2018; Srivastava and Agarwal, 2020). Regarding the general working population, health problems coupled with organizational outcomes of WB imply a rise in absenteeism and staff turnover, and subsequent economic costs for organizations (Milczarek, 2010; Hershcovis et al., 2016).

Thus, the fact of the job being low socioeconomic status and linked to the feminine sphere (due to the tasks involved in the job) might exacerbate the risk of violence and harassment of HHs. Given the characteristics of HHs and the strong effects that SHaW and WB might have at several levels (human rights, health, emotional, economic, organizational), this study wants to fill the existing gap in the literature regarding SHaW and WB experiences of HHs and their prevalence. Hence, the aim of this study was (i) to explore experiences and perceptions of SHaW and WB among HHs in the Balearic Islands and (ii) to describe and estimate the prevalence of SHaW and WB among HHs in the Balearic Islands.

## 2 Materials and methods

This is a mixed-methods study consisting of two distinct phases, qualitative and quantitative, conducted in the primary healthcare setting in the Balearic Islands. This study is part of a wider project, “Hotel Housekeepers and Health,” which is aimed at exploring the hotel housekeeping job and HHs’ health problems, as well as improving HHs’ quality of life and health.

The qualitative study was carried out between February and June 2018. Ten semi-structured interviews were conducted with key informants, and six focus groups (FG) were held with HHs. Taking into account all the information generated and analyzed in the qualitative study, the quantitative study took place between November 2018 and February 2019.

### 2.1 Participants

#### 2.1.1 Qualitative study

HHs participating in FGs were recruited through purposive sampling. General practitioners in different healthcare centers identified potential participants according to sociodemographic and labor variables and informed them about the research. This was the most feasible and effective way to contact them and obtain their participation. Afterward, researchers contacted and invited them to participate in FGs and set the date. Selection criteria included being 18 years of age or older and having worked as a HH during the previous season (2017). Additionally, profiles regarding different variables—age, years working as a HH, hotel star rating, and kind of contract (permanent, temporary, or recurring-seasonal employment contract)—were included to ensure generating rich information. Key informants were selected through purposive sampling to obtain different perspectives and rich information about the HH job.

#### 2.1.2 Quantitative study

HHs who were at least 18 years old, had health coverage in the Balearic Public Health System, worked as HHs during 2018, and were willing to participate in this study were included after signing the informed consent. Those with language barriers to understand the informed consent, the survey, and the questions in FG and interviews in Spanish were excluded.

### 2.2 Sample and data collection

#### 2.2.1 Qualitative study

Empirical material was collected through FGs with HHs—performed in different healthcare centers—and semi-structured interviews of key informants. Four FGs were held in Mallorca, one in Menorca, and one in Ibiza; so each participant attended the FG taking place in the primary health center closest to their home. FGs ranged from 60 to 90 min and interviews from 25 to 80 min, all of which were conducted by the first author. Interviews were recorded digitally, and FGs were video-recorded as well. Data collection was undertaken until saturation of the information was reached.

Thirty-four HHs participated in FGs—between four and eight in each one. A total of 64 HHs were invited: 20 of them refused to participate, and although 10 had initially agreed, in the end, they did not attend the FG. HHs participating in FGs did not receive any financial compensation, but they did receive a small “thank you” gift after their participation (i.e., a bottle of extra-virgin olive oil).

Sociodemographic characteristics of FG participants are displayed in Table 1 and key-informant profile in Table 2.

**Table 1:** Sociodemographic characteristics of participants in the focus groups.

Age (*n* = 34)	
X̄	50 years
SD	10 years
<30	5.9%
30 to 39	5.9%
40 to 49	23.5%
50–59	52.9%
>59	11.8%
Years working as HHs (*n* = 34)	
X̄	19.5 years
SD	11.5 years
<10	17.6%
10–14	20.6%
15–24	32.4%
≥25	29.4%
Tenure (*n* = 34)	
Permanent^1^	2.9%
Recurring-seasonal contract^2^	88.2%
Temporary^3^	8.8%
Hotel category (*n* = 34)	
2*	6.1%
3*	45.5%
4*	42.4%
5*	6.1%

X̄, mean.

SD, standard deviation.

^1^HHs working the whole year.

^2^HHs working only some months per year (usually spring, summer, and autumn), but the company commits to hiring them again the following year.

^3^HHs with a contract that lasts a pre-established number of months.

**Table 2:** Codes and key-informant profiles of interviewees.

Code	Key-informant profile
HHi1	HH belonging to a union
HHi2	HH belonging to a union
HHi3	HH belonging to a HH association
HHi4	HH belonging to a HH association
SUP	HH supervisor
GP	General practitioner in a coastal practice
PInsp	Physician in public inspection service
HRDir	Director of human resources (HR) in a hotel chain
Prev	Head of the occupational risk prevention service in a hotel chain
OHealth	Head of occupational health department in a hotel chain

#### 2.2.2 Quantitative study

An initial list of about 13,000 possible HHs was available from the Balearic Health Services. The sample had to reach 978 HHs to estimate population parameters with a 3% precision and a confidence of 95%. We foresaw including 1,115 HHs with 10% of losses; therefore, for each HH selected, three other HHs were identified—with the same age and from the same area—who could be selected as a replacement.

Health professionals (nurses) were trained to conduct the researcher-administered survey and were put in charge of recruiting HHs. Survey administration lasted for 1 h approximately and was carried out in the HHs’ primary healthcare centre.

We enrolled 1,043 HHs: 773 in Mallorca, 89 in Menorca, 137 in Ibiza, and 44 in Formentera. Table 3 shows the sociodemographic, individual, and labor characteristics of the HHs included in the sample.

**Table 3:** Sociodemographic, individual, and labor characteristics.

	Median	IQR
Age (years)	43.3	36.0–51.02
Years working as HHs	8.0	4.0–15.0
Months worked previous season	7.0	6.0–8.0
Hours worked per week	40.0	40.0–40.0
Number of rooms cleaned/day	18.0	15.0–22.0
	*n* **%**	**95% CI**
Nationality (*n*=1,043)		
Spanish	563 (54.0)	50.9–57.0
Double nationality	182 (17.4)	15.2–19.9
Latin America	169 (93.9)	89.2–96.6
Africa	9 (5.0)	2.6–9.4
Asia	2 (1.1)	0.3–4.4
Foreign	298 (28.6)	25.8–31.4
Latin America	131 (44.6)	38.9–50.3
Africa	89 (30.3)	25.3–35.8
Asia	2 (0.7)	0.2–0.3
European non-EU countries	10 (3.4)	1.8–6.2
EU-countries	62 (21.1)	16.8–26.2
Level of studies (*n*=1,041)		
Illiterate/ primary incomplete	34 (3.3)	2.3–4.5
Compulsory education (primary and secondary)	591 (58.6)	53.7–59.8
Post-compulsory secondary education	98 (28.6)	25.9–31.5
University	118 (11.3)	9.5–13.4
Type of contract (*n*=1,016)		
Permanent	63 (6.2)	4.8–7.9
Recurring-seasonal contract	551 (54.2)	51.1–57.3
Temporary	402 (39.6)	36.5–42.6
Type of establishment (*n*=1,043)		
Hotel	625 (59.9)	56.9–62.9
Apart-hotel	351 (33.7)	30.8–36.6
Rural hotel	42 (4.0)	2.9–5.4
Others	25 (2.4)	1.6–3.5
Hotel category		
1*	9 (1.0)	0.4–1.8
2*	37 (4.0)	2.8–5.4
3*	210 (22.5)	19.9–25.3
4*	574 (61.5)	58.3–64.7
5*	103 (11.0)	9.1–13.2

*Hotel category in stars.

### 2.3 Variables

#### 2.3.1 Qualitative study

Based on the literature review, we developed a script to explore the areas identified as relevant and approach them in a similar way across all FGs and interviews (see Table 4). The initial script was completed as the data collection was progressing. The areas approached in FGs and interviews were the characteristics and organization of the HHs’ work; positive and negative aspects of the HHs’ job; equipment and materials available; relationships between hotel workers and between HHs; stress factors; SHaW and WB; and health problems. This study focused on the analysis of the information related to SHaW and WB.

**Table 4:** Sample questions from the script of interviews and focus groups with hotel housekeepers (qualitative study).

How is a working day of a hotel housekeeper? How would you describe the relationship among co-workers? And with supervisors and managers? Do you have any health problem? Have you ever received contemptuous comments towards you or towards the work you had done by a client or co-worker? If this is the case, how did you react? Did you report it to a supervisor? Have you ever suffered sexual harassment from a client or co-worker? If this is the case, how did you react? Did you report it to supervisor? What was his/her response? How did this response make you feel?

#### 2.3.2 Quantitative study

##### 2.3.2.1 Dependent variables

SHaW and WB were measured through seven questions inspired by the Leymann Inventory of Psychological Terror Scale (Leymann, 1990) and Cisneros Scale (Fidalgo and Piñuel, 2004). The questions were as follows: “Check the corresponding box if any of the following situations have occurred at your workplace: I. personal or professional scorn; II. feeling ignored or invisible; III. verbal intimidation (threats, raised voice, yelling); IV. malicious and humiliating comments; V. excessive supervision (schedules, work, strict control over work); VI. physical threats; VII. indecent sexual advances or propositions.” () answers were “never,” “a few times a year,” “once a month or less,” “a few times a month,” “once a week,” “several times a week,” and “every day.”

These variables were dichotomized according to Leymann’s statistical definition concerning the frequency by which harassment actions occurs (Leymann, 1990); it was assumed that all participants who reported at least once a week were actually suffering from SHaW or WB. Hence, response options “never,” “a few times a year,” “once a month or less,” and “a few times a month” were grouped as “non-harassed,” and “once a week,” “several times a week,” and “every day” were grouped as “harassed.”

##### 2.3.2.2 Independent variables

Sociodemographic variables include age, nationality (Spanish, double nationality, or other), and level of studies.

Labor variables include years working as HHs, months worked during the previous tourist season, hours worked per week, number of rooms cleaned per day, type of contract (permanent, recurring-seasonal, or temporary), accommodation type (apartment, hotel, etc.), and hotel category.

Level of stress at work was measured with the question, “Globally and taking into account the conditions in which you carry out your work, indicate how you consider the stress level of your work on a scale from 1 (very stressful) to 7 (not at all stressful).”

Satisfaction with the job was measured with the question, “Taking into account the characteristics of your job, indicate to what extent you consider your job as satisfactory on a scale from 1 (not satisfactory at all) to 7 (very satisfactory).”

Satisfaction with the salary was measured through the question, “To what extent are you satisfied with your salary? Please, circle the number that describes how you feel. To do so, use the following response scale.” Answers ranged from 1 (lowest level of satisfaction) to 7 (highest level of satisfaction).

Self-rated health: on the day the researcher administered the survey, participants were asked to rate their overall health on a 0–100 vertical visual analog scale taken from the EuroQoL-5D-5L, a generic instrument for describing and valuing health (Herdman et al., 2001).

Social support was measured by DUKE-UNC-11 (Broadhead et al., 1988), an 11-item questionnaire to assess functional elements of social support (including confident and affective support) validated in the Spanish population (de la Revilla Ahumada et al., 1991; Bellón Saameño et al., 1996). A sample item was “Do you receive visits from your friends and relatives?.” Each item is valued on a 5-point scale (ranging from 1 “far less than I would like” to 5 “as much as I would like”). A final score ranging from 5 to 55 is obtained; 32 points or below correspond to low social support, and over 32 points correspond to adequate social support (Bellón Saameño et al., 1996).

### 2.4 Data analysis

#### 2.4.1 Qualitative study

FGs and interviews were transcribed literally. An alphanumeric code was assigned to each HH to guarantee confidentiality but also to be able to identify the contributions of each person. Each contribution of FG participants was identified by “HH” (meaning ‘hotel housekeeper’) and two numbers separated by a dot: the first number corresponding to the FG (ranging from 1 to 6) and the second number pertaining to the individual who made the contribution. A code was also assigned to key informants to guarantee their confidentiality (see Table 2).

The contents of FGs and interviews were analyzed jointly, for the purpose of identifying both similarities and differences in the narratives. Thematic analysis was undertaken following the steps established by Braun and Clarke (2012). First, a code tree (Table 5) was elaborated according to the objectives of the research and the reading of some FG transcriptions. This code tree was checked by a second researcher. To guarantee internal validity, both researchers encoded and analyzed the transcriptions separately. Finally, analysis of each code was discussed, and conclusions were agreed. Software NVivo11 was used to assist this analysis.

**Table 5:** Code tree regarding sexual harassment at work and workplace bullying.

Sexual harassment	
	Behaviors involving sexual harassment
	People who perpetrated sexual harassment
	Reactions to sexual harassment
	Response obtained from hotel management
	Assessment of the response obtained from hotel management in cases of sexual harassment
Bullying	
	Behaviors involving bullying
	People who perpetrated bullying
	Reactions to bullying
	Response obtained from hotel management
	Assessment of the response obtained in cases of bullying

#### 2.4.2 Quantitative study

Categorical variables (such as nationality, level of education, type of contract, etc.) are presented in absolute numbers along with percentages and 95% confidence intervals (95% CI), while quantitative variables (years working as HHs, months worked/year, etc.) are presented as medians and interquartile range (IQR).

Statistical analysis using SPSS for Windows version 23.0 was used for descriptive analysis and estimations of 95% CIs. Bivariate analysis was used to assess the relationship between sociodemographic, individual, and labor variables and the prevalence of SHaW and WB. The chi-square test and Mann–Whitney U-test were calculated. *P*-values of less than 0.05 were considered statistically significant (two-sided tests).

### 2.5 Ethics approval

The study was approved by the Balearic Islands Research Ethics Committee (IB3738/18 PI). An information sheet and informed consent were given to the participants before undertaking the FG or interview and before being enrolled in the quantitative study. Signed agreement of the forms was compulsory to participate.

## 3 Results

### 3.1 Qualitative study

Regarding SHaW, when the general and open question was posed (“Have you experienced sexual harassment in your workplace?”), HHs spontaneously gave a negative answer, mentioning that they had not experienced it. However, when the moderator gave some examples of situations of sexual harassment, participants in all FGs and HHs interviewed as key informant reported to have experienced them either personally or by a co-worker. However, the most severe situations were perceived as unusual by HHs.

*Moderator (M): Guests that are naked when you go into the room…*

*HH 3.1: Oh well, yeah, that yes.*

*HH 3.2: That yes, that’s why we see it as normal.*

According to FGs and interviews, all sexual harassment situations were guest-initiated. From more to less common, the following situations were mentioned by HHs:

- Guests were naked when HHs went into the room to clean it. HHs deemed this kind of sexual innuendo or indecent exposure as mischief.

*HH 6.5: And it’s happened to me, that you knock the room and the man’s naked and you say, “Oh! Sorry, I’ll come back later.” And they say to you, “No, come in, come in.” And he’s naked.*

*HH 4.5: I had a guest who always waited until his wife went to the swimming pool and when I was opposite, well he went inside and got undressed. And then, he was waiting for me to knock on the door. When I knocked on the door, he did this. He would get naked like this. Every day he did the same.*

- Guests chasing HHs through the hotel.

*HH 6.8: Last year we had one man who chased the housekeepers, but the hotel manager was very quick.*

*M: Because, what do you do in those cases?*

*HH 6.1: Warn them not to make up that room.*

*HH 6.2: Or the executive housekeeper goes up, so the girl does not have to go alone, or she sends another co-worker, so they make up the room together.*

- Unwanted sexual comments or propositions from guests.

*HHi4: Let us see, sexual harassment from guests, yes. More than from co-workers […]. And coming in drunk and asking you to masturbate them […]. This year I had one workmate that this happened to with a client. Opening up to her naked. The guest came out, he did not want any cleaning, but he propositioned her, asking her if she could masturbate him, and she came to me crying to tell me about it and I said to her, “Come on, we are going down to tell the executive housekeeper.” Her fear was that she’d be sacked. The thing is, on top of everything, you blame yourself. That’s still happening nowadays.*

- Guests who closed the door once the HH was inside the room.

*HH 4.7: I took advantage that they were on their way out, I told him if I could do the room for them, they said yes, they went to the pool. When I realized, I’d made the beds and everything. I went into the bathroom, you know, a small bathroom, the door. The guy came in, I saw him come in, “Oh, hello.” As I’d seen him go out, I knew he was the guest in this room. When I realized, he had me cornered in the bathroom, touching my bum, and speaking to me in German, which I did not understand.*

- Unwanted touching.

*SUP: Sometimes a guest wanted to go too far. If the guest is drunk, for example. But well, no. This issue cannot be judged as harassment (…)*

*M: What happened, for example?*

*SUP: Well, a guest arrived and gave a little slap on the backside, for example.*

*M: But there are cases…*

*SUP: Very, very, very, very isolated.*

The HHs’ attitude was to normalize this kind of behaviors, giving little importance to these situations or even not acknowledging them as SHaW.

*HH 6.1: Harassment, not harassment, no. But have not you ever had the case of someone coming out naked? […]. Or you are on a balcony and there’s someone naked on the next-door balcony. Or they open the door to you stark naked and they say, ‘come in, come in’. ‘Later, I’ll come when you are not in’.*

*HH 6.7: Ah, that has happened, yes.*

When faced with these situations, HHs reported being alone because there was no co-worker nearby they could ask for help. HHs explained that when they reported this kind of situation to the supervisor, it was their word against the guests. However, HHs stated that once they reported it, despite not opening a formal claim against the client, hotel management and the supervisor gave a response to that situation, such as talking to the guest, not cleaning the room during the whole stay, or going in pairs to clean the room.

*HH 2.2: About co-workers, yes. A young girl, erm, she was going to do the room and the guest closed the door on her. And – well, it happened two days, and on the third the executive housekeeper went with her.*

Despite this quite permissive response, HHs valued it positively because they felt supported. On many occasions, HHs also reacted to these sexual harassment situations with humor, above all, in the situations in which the client was naked inside the room when they entered to clean it.

*HH 2.1: Now, for them to open the door to the room while naked, that does happen. I say, “Well, I’ll come back at another time.” They’re completely unconcerned (she laughs). That’s why I laugh, because “shame on them.”*

Finally, participants reported that HHs received advice in training courses organized by hotels about how to avoid situations of sexual harassment by guests.

*HRDir: Some nonsense from a guest… But nothing relevant, no. From guests, they (HHs) always have to be very careful. Of course, a lot of emphasis is placed on always leaving the door open, “Oh, I’m staying inside, you can close the door.” No, no, the door always open, there must be communication….*

The open question about WB was “Have you been bullied by any co-worker or have you received any humiliating comments of scorn from a guest?” Although the first answer was a negative one, some HHs in the FGs reported not being well-treated by the executive housekeepers (their immediate supervisor), not receiving recognition for work well-done and perceiving that some guests looked down on them (although HHs perceived that most guests treated them with respect).

*HH 5.3: people are very pleasant, but it’s true that some people come and look down on you as if they were saying…I’m above and you are below.*

*HH 6.2: Not contempt, but the guests do usually treat you badly sometimes.*

*HH 6.8: I’ve seen an executive housekeeper call us all lazy and slobs. Because we did not know how to clean.*

Despite this, in the FGs, HHs only reported one serious case of bullying by a co-worker, in which the hotel reacted immediately by replacing the person who was bullying. While HHs did not report cases that fitted into the definition of bullying in the FG, the interviews revealed that WB situations were quite common. Probably, the fact that the HHs interviewed were members of HH associations or unions made them more familiar with the term of WB and more exposed to receiving information on bullying cases. Moreover, the interview technique makes it possible to delve further into topics and explanations than FGs; so, interviewees were able to better explain the topics.

*HHi2: They have to prepare them [the managers or executive housekeepers] to be tactful, because that’s another thing there is; they treat people like shit, in capital letters, do not they? Honestly. And that’s the saddest thing of all. That you go to work and they are constantly disrespecting you, because I’ve had loads of problems with that, not personally with me (…). But I know many people who have had lots of problems and what happens is that they do not want to say anything.*

*HHi4: Then you have workplace bullying; they give you more work, they send you less help. Or they give you a lower rank, the places that are furthest away …*

Key informants working in hotels described these cases as unusual. The occupational health manager of a hotel explained that hardly any cases of WB occurred in the hotel. Despite this, he did report one case of bullying and how the existing protocol was applied.

*OHealth: In some cases, there might have been, both vertical and transversal. What happens is that now, when there’s a case of bullying, we already have a protocol.*

### 3.2 Quantitative study

Table 6 displays the results of the prevalence of SHaW and WB situations suffered by HHs.

**Table 6:** Prevalence of workplace bullying and sexual harassment at work.

	Never *n* (%) *95% CI*	A few times a year *n* (%) *95% CI*	Once a month or less *n* (%) *95% CI*	A few times a month *n* (%) *95% CI*	Once a week *n* (%) *95% CI*	Several times a week *n* (%) *95% CI*	Every day *n* (%) *95% CI*	TOTAL *n* (%)
Excessive supervision	560 (53.7) *50.6–56.8*	83 (8.0) *6.4–9.8*	41 (3.9) *2.8–5.3*	69 (6.6) *5.2–8.3*	46 (4.4) *3.2–5.8*	104 (10.0) *8.2–12.0*	140 (13.4) *11.4–15.6*	1.043 (100)
Feeling ignored or invisible	579 (55.6) *52.5–58.6*	106 (10.2) *8.4–12.2*	62 (6.0) *4.6–7.6*	117 (11.2) *9.4–13.3*	32 (3.1) *2.1–4.3*	98 (9.4) *7.7–11.3*	48 (4.6) *3.4–6.1*	1.042 (100)
Personal or professional scorn	511 (49.0) *45.9–52.1*	185 (17.7) *15.5–20.2*	81 (7.8) *6.2–9.6*	121 (11.6) *9.7–13.7*	31 (3.0) *2.0–4.2*	80 (7.7) *6.1–9.5*	34 (3.3) *2.3–4.5*	1.043 (100)
Malicious and humiliating comments	633 (61.0) *58.0–54.0*	121 (11.7) *9.8–13.8*	74 (7.1) *5.6–8.9*	84 (8.1) *6.5–9.9*	28 (2.7) *1.8–3.9*	64 (6.2) 4.8–7.8	33 (3.2) *2.2–4.4*	1.037 (100)
Verbal intimidation	671 (64.4) *61.4–67.3*	126 (12.1) *10.2–14.2*	56 (5.4) *4.1–6.9*	65 (6.2) *4.8–7.9*	34 (3.3) *2.3–4.5*	63 (6.0) *4.7–7.7*	27 (2.6) *1.7–3.7*	1.042 (100)
Physical threats	1.007 (96.7) *95.5–97.7*	19 (1.8) *1.1–2.8*	4 (0.4) *0.1–1.0*	2 (0.2) *0.0–0.7*	2 (0.2) *0.0–0.7*	2 (0.2) *0.0–0.7*	5 (0.5) *0.2–1.1*	1.041 (100)
Indecent sexual advances or propositions	954 (91.8) *90.0–93.4*	51 (4.9) *3.7–6.4*	13 (1.3) *0.7–2.1*	13 (1.3) *0.7–2.1*	0 *0–0.4*	4 (0.4) *0.1–1.0*	4 (0.4) *0.1–1.0*	1.039 (100)

Proportions of HHs who had suffered physical threats or sexual harassment at least once a week were 0.9 and 0.8%, respectively. In total, 39% HHs reported suffering the bullying behaviors included in the questionnaire at least once a week, in particular, excessive supervision (27.8%), feeling ignored or invisible (17.1%), personal or professional scorn (14%), receiving malicious and humiliating comments (12.9%), and verbal intimidation (11.9%).

The results of the analysis regarding the association between age, nationality, hotel category, and type of contract, and the different situations of SHaW and WB are shown in Table 7. By age, those who suffered bullying behavior at least once a week were statistically significantly younger (median = 42.4; SD 10.3) than those who did not (median = 43.8; SD = 10.0) (*p* = 0.033). A more detailed analysis revealed that significant differences by age were related only to personal or professional scorn, with victims turning out to be younger (median = 40.9; SD = 10.1) than non-victims (median = 43.6; SD = 10.1) (*p* = 0.002).

**Table 7:** Results of the association between workplace bullying and sexual harassment at work and individual and labor variables.

	Professional or personal scorn			Feeling ignored or invisible			Verbal intimidation			Malicious and humiliating comments			Excessive supervision			Physical threats			Indecent sexual advances or propositions		
	At least once a week	Less than once a week	*p*-value	At least once a week	Less than once a week	*p*-value	At least once a week	Less than once a week	*p*-value	At least once a week	Less than once a week	*p*-value	At least once a week	Less than once a week	*p*-value	At least once a week	Less than once a week	*p*-value	At least once a week	Less than once a week	*p*-value
Age X̄ (SD)	40.9 (10.0)	43.6 (10.1)	0.002	43.0 (10.1)	43.3 (10.1)	0.741	42.4 (9.1)	43.3 (10.2)	0.281	41.8 (10.0)	43.5 (10.1)	0.101	42.6 (10.3)	43.5 (10.1)	0.222	40.3 (11.0)	43.3 (10.1)	0.577	40.1 (5.4)	43.2 (10.1)	0.343
	%	%	*p*-value	%	%	*p*-value	%	%	*p*-value	%	%	*p*-value	%	%	*p*-value	%	%	*p*-value	%	%	*p*-value
Nationality																					
Spanish	60.0	53.0	0.282	59.6	52.9	0.267	56.6	53.6	0.828	56.0	53.9	0.816	56.6	53.0	0.213	66.7	53.9	0.783	62.5	53.9	0.879
Double nationality	14.5	17.9		15.2	17.9		16.1	17.6		15.2	17.4		14.1	18.7		11.1	17.5		12.5	17.6	
Foreign	25.5	29.1		25.3	29.2		27.4	28.8		28.8	28.6		29.3	28.3		22.2	28.6		0.7	28.5	
Hotel category																					
1*	1.5	0.9	0.081	1.2	0.9	0.135	0.9	1.0	0.027	0.9	1.0	0.097	0.0	1.3	0.114	0.0	1.0	0.281	0.0	1.0	0.613
2*	2.2	4.3		1.8	4.4		2.6	4.2		2.6	4.2		2.3	4.6		0.0	4.0		0.0	4.0	
3*	15.6	23.7		16.9	23.8		11.3	24.1		13.2	23.7		21.4	22.9		0.0	22.8		0.0	22.7	
4*	65.2	60.9		67.5	60.2		71.3	60.1		71.9	60.2		65.4	60.0		1.4	61.1		85.7	61.3	
5*	15.6	10.3		12.7	10.7		13.9	10.6		11.4	10.9		10.9	11.1		0.0	11.2		14.3	11.1	
Type of contract																					
Permanent	7.1	6.1	0.695	6.9	6.1	0.699	5.0	6.4	0.364	3.4	6.6	0.040	4.2	7.0	0.081	0.0	6.3	0.791	0.0	6.3	0.686
Recurring-seasonal	51.1	54.7		56.3	53.9		49.6	54.9		47.1	55.1		51.6	55.3		57.1	54.3		50.0	54.3	
Temporary	41.8	39.2		36.8	40.1		45.4	38.7		49.6	38.3		44.2	37.8		42.9	39.4		50.0	39.4	

Statistically significant differences were found for verbal intimidation by hotel category (*p* = 0.027), such that higher percentages of victims worked in 4- and 5-star hotels, and for malicious and humiliating comments by type of contract, whereby more victims were among those with a temporary contract (*p* = 0.040). No statistically significant differences were found regarding nationality.

The results of the association between SHaW and WB and self-rated health, stress at work, satisfaction with job and salary, and social support are displayed in Table 8. There is a statistically significant association between poorer self-rated health and suffering from professional or personal scorn (*p* ≤ 0.001), feeling ignored or invisible (*p* ≤ 0.001), verbal intimidation (*p* ≤ 0.001), receiving malicious and humiliating comments (*p* = 0.001), and excessive supervision (*p* = 0.001).

**Table 8:** Results of the association between workplace bullying and sexual harassment at work and self-rated health, stress at work, satisfaction with job and salary, and social support.

	Professional or personal scorn			Feeling ignored or invisible			Verbal intimidation			Malicious and humiliating comments			Excessive supervision			Physical threats			Indecent sexual advances or propositions		
	At least once a week	Less than once a week	*p*-value	At least once a week	Less than once a week	*p*-value	At least once a week	Less than once a week	*p*-value	At least once a week	Less than once a week	*p*-value	At least once a week	Less than once a week	*p*-value	At least once a week	Less than once a week	*p*-value	At least once a week	Less than once a week	*p*-value
Self-rated health X̄ (SD)	66.6 (21.2)	73.3 (18.4)	≤0.001	67.3 (21.5)	73.4 (18.2)	≤0.001	65.4 (19.7)	73.3 (18.7)	≤0.001	66.6 (20.0)	73.2 (18.7)	0.001	69.1 (19.9)	73.6 (18.5)	0.001	60.0 (21.2)	72.5 (18.2)	0.115	70.6 (19.7)	72.4 (19.0)	0.809
Level of stress at work X̄ (SD)	6.0 (1.4)	4.8 (1.8)	≤0.001	5.9 (1.4)	4.7 (1.8)	≤0.001	6.0 (1.4)	4.8 (1.8)	≤0.001	6.0 (1.4)	4.8 (1.8)	≤0.001	5.8 (1.6)	4.6 (1.8)	≤0.001	5.8 (1.6)	4.9 (1.8)	0.145	6.6 (0.7)	4.9 (1.8)	0.009
Satisfaction with the job X̄ (SD)	4.2 (2.1)	5.0 (1.8)	≤0.001	4.2 (2.1)	5.1 (1.8)	≤0.001	4.2 (2.2)	5.0 (1.8)	≤0.001	4.4 (2.1)	5.0 (1.8)	≤0.001	4.3 (2.0)	5.2 (1.7)	≤0.001	4.1 (2.2)	4.9 (1.8)	0.294	4.0 (2.0)	4.9 (1.8)	0.228
Satisfaction with the salary X̄ (SD)	3.3 (1.9)	4.2 (2.0)	≤0.001	3.3 (2.0)	4.3 (2.0)	≤0.001	3.3 (2.0)	4.2 (2.0)	≤0.001	3.5 (2.0)	4.2 (2.0)	0.001	3.5 (2.0)	4.3 (2.0)	≤0.001	2.4 (1.9)	4.1 (2.0)	0.029	2.6 (2.2)	4.1 (2.0)	0.100
	%	%	*p*-value	%	%	*p*-value	%	%	*p*-value	%	%	*p*-value	%	%	*p*-value	%	%	*p*-value	%	%	*p*-value
Social support																					
Normal	88.7	94.5	0.009	91.4	94.1	0.185	86.8	94.6	0.001	88.5	94.4	0.012	89.6	95.3	0.001	88.9	93.8	0.546	57.1	93.9	≤0.001
Low	11.3	5.5		8.6	5.9		13.2	5.4		11.5	5.6		10.4	4.7		11.1	6.2		42.9	6.1	

Furthermore, HHs suffering from professional or personal scorn (*p* ≤ 0.001), feeling ignored or invisible (*p *≤ 0.001), verbal intimidation (*p* ≤ 0.001), receiving malicious and humiliating comments (*p* ≤ 0.001), excessive supervision (*p* ≤ 0.001), and sexual harassment (*p* = 0.009) reported higher levels of stress at work.

Participants who reported professional or personal scorn (*p* ≤ 0.001), feeling ignored or invisible (*p* ≤ 0.001), suffering from verbal intimidation (*p* ≤ 0.001), receiving malicious and humiliating comments (*p* ≤ 0.001), and excessive supervision (*p* ≤ 0.001) reported lower levels of satisfaction with their job.

HHs who suffered from personal scorn (*p* ≤ 0.001), feeling ignored or invisible (*p* ≤ 0.001), suffering from verbal intimidation (*p* ≤ 0.001), receiving malicious and humiliating comments (*p* = 0.001), excessive supervision (*p* ≤ 0.001), and physical threats (*p* = 0.029) reported lower levels of satisfaction with the salary.

HHs who suffered from professional or personal scorn (*p* = 0.009), verbal intimidation (*p* = 0.001), malicious and humiliating comments (*p* = 0.012), excessive supervision (*p* = 0.001), and sexual harassment (*p* ≤ 0.001) reported low social support.

There was a statistically significant association between suffering WB and poorer self-reported health, higher levels of stress at work, lower levels of satisfaction with the job and salary, and lower levels of social support (Table 8). Additionally, HHs suffering from SHaW reported higher levels of stress at work and lower social support.

## 4 Discussion

This mixed-methods study aimed to explore the situation of HHs in the Balearic Islands regarding SHaW and WB. HHs are close to being a 100% feminized occupational group with a precarious job, with some features of the job increasing the risk of SHaW and WB (i.e., working alone, working in contact with the public, and working in intimate spaces) (ILO, 2017).

The results of the qualitative study point out that the participants in FGs did not self-label themselves as being sexually harassed when the question posed was generic, except for the most severe situations. However, when given examples about behaviors that constitute SHaW, most of them acknowledged having experienced some of them. Although most participants in FGs had been sexually harassed, they normalized this kind of situations (above all, less severe situations); they did not associate them with the term ‘sexual harassment’, and these events were seen as unimportant, in line with the results of Onsøyen et al. (2009). They were also perceived as normal and unquestioned, and part of their daily work (Guerrier and Adib, 2000; ILO, 2017). Although we did not find studies including HH self-labeling, other studies found that a significant proportion of women who reported an experience associated with sexual harassment did not label it as sexual harassment (Orchowski et al., 2013; Buchanan et al., 2018).

This might partly explain why they reacted to these less severe situations without getting angry and with humor, a strategy to respond to sexual harassment also reported by HHs in other studies (Guerrier and Adib, 2000; Kensbock et al., 2015, 2016). This kind of strategy allows HHs to reject a guest’s advances without offending them while maintaining a show of respect (Kensbock et al., 2015). More severe situations were perceived as unusual. Despite this, HHs and key informants acknowledged that training courses offer advice to avoid sexual harassment by guests, such as fixing room doors open while inside the room cleaning, in accordance with previous studies (Hunter and Watson, 2006; Kensbock et al., 2015). This indicates that everyone in the sector is aware of this risk.

Narratives of HHs showed no hesitation in reporting the most severe episodes of sexual harassment to the executive housekeeper or to managers and key informants reported that there were protocols for action in case of a sexual harassment incident. Kensbock et al. (2015) also explored the protection given in the workplace to HHs in case of sexual harassment and found that although protocols existed, these were not always appropriate for reporting. Kensbock et al. (2015) reported that sometimes managers or supervisors were not able to properly assess the severity of a certain situation and HHs interviewed also highlighted the power guests can exert through negative evaluations in the satisfaction surveys. Contrary to Kensbock et al.’s results, HHs in our study assessed the response given by hotel managers as reasonable, a feature that might lead HHs to more easily report these events.

The little importance attributed to, and low awareness of, sexual harassment events at work reported in the qualitative study is consistent with the results of the quantitative study (i.e., 0.8% of the participants reported indecent sexual advances or propositions at least once a week). This percentage is slightly different from the results of the European Working Conditions Survey (EWCS); in Spain, 0.6% women reported sexual harassment, while 1.7% reported unwanted sexual attention (Eurofound, 2015). Although some studies performed in the general working population showed a positive relationship between precarious employment and SHaW (Torres et al., 2016; Reuter et al., 2020) and HHs are in precarious employment, our results do not point to a higher prevalence of sexual harassment among them. This might be explained in part by the difficulty HHs have in labeling certain situations as SHaW.

Given that HHs did not identify some situations mentioned in FGs as SHaW, we consider that using the behavioral experience method—whereby a range of behavioral experiences is presented to participants—might better reflect the prevalence and experiences of SHaW than self-labeling methods—which consist of asking participants whether they have been sexually harassed (Orchowski et al., 2013; Buchanan et al., 2018). Given these results, future research might consider including both methods.

An explanation for the normalization of SHaW lies in the symbolic structure of patriarchy, that is, the values, ideas, and social definitions that uphold society and make it work (Galarza et al., 2016). The symbolic structure of patriarchy associates women with nature, sexuality, and feelings, while men are related to culture and rationality—traits that are considered superior—and try to impose a model of femininity in which women are depicted as objects—and thus, as inferior—at the service of masculine power, and there to satisfy their sexual desire (Cobo Bedia, 2015). As MacKinnon (1979) stated, sexual harassment contributes to keeping women feeling in an inferior social status. This idea also explains the behavior of clients who initiate sexual approaches: Men understand that women are available to them to satisfy their sexual needs and wishes. To sum up, the fact that hotel housekeeping is socially considered unskilled (Nimri et al., 2020) and that job roles are related to domestic tasks emphasizes their position of inferiority with respect to customers; this, coupled with the fact that the job is performed in private spaces (i.e., the room) and that part of this job consists of satisfying guests’ needs, might lead to HHs being at risk of being more vulnerable to sexual harassment.

Both key informants and FG participants perceived more extreme situations of SHaW as less frequent than mild situations. These perceptions do not completely agree with the results of Nimri et al. (2020), whereby human resource managers and executive housekeepers reported SHaW events as rare.

The results of the quantitative study highlighted that HHs who were sexually harassed mentioned higher levels of stress at work and lower social support. Despite this, narratives of HHs indicated a positive assessment of the response of hotel management in sexual harassment cases, situation that might be understood as social support at work. This apparent contradiction between the quantitative and qualitative results might be explained, among others, because the DUKE-UNC-11 questionnaire is not specific for measuring social support at work. Furthermore, we have to take into account that narratives of HHs might be influenced by other HHs participating.

A positive relationship between being sexually harassed and psychological distress has also been identified in other studies (Hutagalung and Ishak, 2012; Holland and Cortina, 2016). Sigursteinsdottir and Karlsdottir (2022) also found that those who had been sexually harassed in their workplace reported lower levels of social support at work; furthermore, Anwar (2022) found that social support mediated the effects of SHaW on the victim.

The relationship between SHaW, social support, and job satisfaction has been studied in other occupational groups. Holland and Cortina (2016) reported a relationship between SHaW and lower job satisfaction among workers of several industries, as well as Hutagalung and Ishak (2012) in their study among female university employees. Furthermore, the study by Alrawadieh et al. (2021)reported a negative relationship between SHaW, organizational social support, and job satisfaction among Turkish female tourist guides.

Regarding WB, although the first answer was a negative one—the same as when being asked about sexual harassment—some HHs in the FG mentioned that executive housekeepers did not usually treat them well and they did not recognize their well-done work. Similarly, latina HHs identified different mistreatment behaviors at work, such as verbal abuse, feeling unfairly treated, and unfair work assignments by their supervisors (Hsieh et al., 2017). In the study by Hsieh et al. (2017), origin and ethnicity were deemed by some HHs as triggers of mistreatment; however, origin and ethnicity do not seem an additional risk for WB in our sample.

The qualitative results are in line with the quantitative ones, which revealed that excessive supervision (at least once a week) was the situation most suffered by participants, followed by feeling ignored or invisible. Moreover, HHs participating in FGs perceived that guests looked down on them. In general terms, HHs positively assessed the response given by hotel managers in the presence of severe situations of WB, contrary to the experiences reported in Kensbock et al. (2015). Nevertheless, it is noteworthy that HHs did not report milder situations to hotel management; this might be explained because this kind of behaviors is understood as inherent to the job, similar to the findings of Mathisen et al. (2008) in the restaurant sector, or because milder harassment behaviors might lead to confusion and, thus, are less likely to be reported (Samnani, 2013).

The results of the quantitative study showed that HHs suffering from personal or professional scorn were younger, and there were higher percentages of participants who suffered from malicious and humiliating comments among those with a temporary contract. HHs who suffered from personal or professional scorn, verbal intimidation, malicious and humiliating comments, or excessive supervision reported poorer self-rated health, higher levels of stress at work, lower satisfaction with the job and the salary, and lower social support. Participants who felt ignored or invisible indicated poorer self-rated health, higher stress at work, and lower satisfaction with the job and the salary. The EWCS (Eurofound, 2015) used similar categories regarding WB. This allows for comparing the results for HHs to those for women surveyed in the EWCS sample. Thus, 11.9% of HHs reported verbal intimidation compared to 6.9% of women who reported verbal abuse at work in the EWCS 2015. Almost 1% HHs reported physical threats, while 3.0% women responding to the EWCS 2015 reported threats. More than 10% of HHs indicated receiving malicious and humiliating comments, whereas only 4.4% in the EWCS 2015 reported humiliating behaviors. A study among Spanish nurses (89% female) found an 8% prevalence of weekly or daily WB; most bullying behaviors reported were related to the tasks given and opinions being ignored (Iglesias and Vallejo, 2012). Hence, compared to other working women, some behaviors related to WB are more prevalent among HHs, such as excessive supervision, while others, such as feeling ignored, are more common among working women.

Our results show a positive relationship between WB and stress. Some studies have identified WB as a predictor of stress, affecting both the individual and personal level (Yaman, 2015) and stress at work (Feijó et al., 2019). However, other studies state that stress at work is the predictor of WB (Reknes et al., 2014; Van den Brande et al., 2016), underscoring the idea that stressful working conditions might be an antecedent and an outcome of harassment. The directionality of this relationship has not been well-established (Nielsen and Einarsen, 2018).

Our results revealed a relationship between being harassed and reporting poorer health. These results are in line with the study by Hewett et al. (2018), in which people who had experienced bullying at work reported lower levels of wellbeing. Additionally, the literature review by Nielsen and Einarsen (2018) identified long-term negative consequences on the health of those who had been bullied. Furthermore, Xu et al. (2018) found that bullied people had a higher risk of developing type 2 diabetes, and this association was similar among women and men.

Moreover, we found a positive association between different behaviors involving WB and lower levels of satisfaction with the job and salary. Similar associations were found in other studies in the tourism sector (Mathisen et al., 2008; Ram, 2018).

Furthermore, the results demonstrated that being exposed to WB was negatively related to social support, an association backed up by the findings of other studies (Feijó et al., 2019; Sigursteinsdottir and Karlsdottir, 2022), some of which placed more importance on organizational support than family support (Rossiter and Sochos, 2018). Despite these results, the narratives in the qualitative study indicated that most of the HHs who suffered from a situation of WB assessed the response of hotel management positively. This discrepancy between quantitative and qualitative results might be explained in part because the direction of causality between harassment and social support cannot be established by cross-sectional studies; thus, people with low social support might easily appear isolated, less popular, with less social support, and thus, they may also become victims easier.

### 4.1 Limitations and strengths

The use of FGs might entail some limitations, such as the difficulty in sharing more severe or personal situations of SHaW and WB. This might be fostered by the fact that some participants knew each other. However, this technique did enable us to identify relevant patterns in HHs experiences.

The large size of the sample guarantees the representativeness of the results. Nonetheless, the cross-sectional methodology does not allow the direction of causality to be established between being a victim of SHaW or WB and perceived poorer health status, higher stress at work, lower levels of satisfaction with the job and salary, and lower social support.

Furthermore, SHaW involves more situations than “indecent sexual advances or propositions” (the only item regarding sexual harassment included in the questionnaire). Hence, it is likely that several sexual harassment situations and behaviors were not captured by the questionnaire, such as environmental sexual harassment (i.e., gender jokes creating a hostile or offensive working environment). For this reason, SHaW prevalence might well be under-recorded, and a recommendation for future research would be to use validated and more detailed measures of these phenomena. Another limitation of this study is the impossibility to gather data on organizational factors as antecedents of SHaW given that the HHs studied worked for different hotels and companies, which were not identified.

This study was carried out in Spanish hotels, where the vast majority of the guests are from central Europe. Considering that these cultures are less sexist than others in the world, transcultural studies are needed to delve into the phenomenon of sexual harassment in the hotel industry as a whole. Furthermore, studies in other regions in Spain would be interesting to widen the knowledge about these phenomena in Spain and determine to what extent the situation is similar.

### 4.2 Practical implications

This study underscores the fact that all workers in the hotel industry are aware of the problem of sexual harassment and bullying in their workplaces, but hotels must improve the actions taken to address these workplace health and safety problems. Actions should include broadening the existence of protocols related to SHaW and WB in all hotels. Risk prevention training programs in the hotel industry should include both topics—SHaW and WB—as a key priority. It is also important for hotel managers to receive this kind of training, as well as HHs and other hotel workers.

## 5 Conclusion

In the Balearic Islands, HHs are close to being a 100% feminized occupational group. Their working conditions are precarious, and the job combines several features that put HHs at a higher risk of being victims of SHaW and WB.

The results of our study indicate that the proportion of HHs who indicated having been sexually harassed was quite low; however, in the qualitative study, HHs acknowledged having often experienced the less severe situations of sexual harassment once they had been given some examples. Hence, these results show the high tolerance to less severe expressions of SHaW (i.e., finding a client inside the room naked) and WB (i.e., excessive supervision), as well as difficulties or reluctance in labeling this kind of experiences as such.

The prevalence of different WB behaviors was quite high among HHs working in the Balearic Islands, and some were associated with poorer self-rated health, less satisfaction with the job and the salary, lower social support, and higher levels of stress. Despite this, qualitative methods inform that less severe behaviors were normalized and perceived by HHs as inherent to their job.

Our results show the importance of refining instruments that are able to identify and quantify the prevalence of, above all, sexual harassment. Additionally, a deeper societal change is needed to empower women to label all kinds of sexual harassment behaviors as such, as well as workplace bullying behaviors. This also includes organizations that should be in charge of implementing measures—not only training, but also supporting the victim and breaking down the climate of impunity surrounding sexual harassment—to tackle this kind of gender violence and protect female workers.

## Data availability statement

The datasets presented in this study can be found in online repositories. The name of the repository and accession number can be found at: Zenodo, https://zenodo.org/, DOI: 10.5281/zenodo.8023440.

## Author contributions

XC-A, OB, and JL: conceptualization. XC-A and JL: methodology. XC-A, OB, and MG-B: validation and formal analysis. XC-A and OB: investigation. XC-A and VF-P: writing—original draft preparation. OB, MG-B, VF-P, JL, and XC-A: writing—reviewing and editing. JL and XC-A: project administration. All authors contributed to the article and approved the submitted version.
